# A population based case–control study of association between dietary calcium intake and ulcerative colitis in adults

**DOI:** 10.1038/s41598-022-11597-8

**Published:** 2022-05-12

**Authors:** Zahra Hajhashemy, Parvane Saneei, Ammar Hassanzadeh Keshteli, Hamed Daghaghzadeh, Hamid Tavakkoli, Peyman Adibi, Ahmad Esmaillzadeh

**Affiliations:** 1 Student Research Committee, Isfahan University of Medical Sciences, Isfahan, Iran; 2 Department of Community Nutrition, School of Nutrition and Food Science, Food Security Research Center, Isfahan University of Medical Sciences, PO Box 81745-151, Isfahan, Iran; 3 Department of Medicine, University of Alberta, Edmonton, AB Canada; 4 Isfahan Gastroenterology and Hepatology Research Center, Isfahan University of Medical Sciences, Isfahan, Iran; 5 Obesity and Eating Habits Research Center, Endocrinology and Metabolism Molecular-Cellular Sciences Institute, Tehran, Iran; 6 Endocrinology and Metabolism Research Center, Endocrinology and Metabolism Clinical Sciences Institute, Tehran University of Medical Sciences, Tehran, Iran; 7 Department of Community Nutrition, School of Nutritional Sciences and Dietetics, Tehran University of Medical Sciences, Tehran, Iran

**Keywords:** Nutrition, Nutrition disorders, Inflammatory bowel disease

## Abstract

Limited data are available on the association of dietary calcium intake and ulcerative colitis (UC). We aimed to investigate the relation between dietary calcium intake and UC prevalence in Iranian adults. In this population-based case–control study, diagnosed patients with UC by gastroenterologists that were registered in the Iranian inflammatory bowel disease registry were included as cases. Age and sex-matched healthy controls were selected from Study on the Epidemiology of Psychological, Alimentary Health and Nutrition (SEPAHAN) dataset. Dietary calcium intakes of participants were examined through a validated food frequency questionnaire. We included 327 middle-aged participants (109 cases and 218 controls) in the analysis; 52.1% of them were females. After adjustments for potential confounders, individuals in the third tertile of dietary calcium intake had 92% lower odds of UC, compared to those in the first tertile (OR = 0.08, 95% CI 0.02–0.27). Our analysis based on recommended dietary allowances (RDAs) intake showed that dietary Ca intake deficiency was related to increased odds of UC (OR = 9.5, 95% CI 2.98–30.91). Stratified analysis by gender revealed that these associations were significant in both genders; although the results were stronger in the male population. A Significant decreasing trend was observed for odds of UC in tertiles of dietary calcium intakes, in both males and females. Higher dietary calcium intake was associated with lower UC prevalence in Iranian adults. Inadequate dietary calcium intake was also linked to elevated odds of UC. Further prospective investigations are needed to affirm these findings.

## Introduction

Inflammatory bowel disease (IBD) is an importation intestinal-autoimmune disease that consists of two main types of Crohn's disease (CD) and ulcerative colitis (UC). Diarrhea, fatigue, abdominal pain and cramping, existence of blood in the stool, reduced appetite and unintended weight loss are more prevalent symptoms of IBD which decrease quality of life in patients^1,2^. Prior studies documented that UC is the common type of IBD in the Iranian population; such that, a national survey in Iran estimated that there were 40.67 patients with ulcerative colitis per 100,000 individuals^3^.

Although previous investigations could not exactly determine the etiology of UC, it was suggested that the main cause could be a severe inflammatory response to altered gut microbiota in a genetically susceptible host. Additionally, some environmental risk factors are related to incidence of IBD symptoms, including smoking, stress, lifestyle, and dietary intakes^2,4,5^. It was documented that inappropriate dietary habits and dietary intakes would alter the intestinal microbiota and lead to an inflammatory response in a genetically susceptible host^4^. For instance, the Mediterranean diet, which is rich in anti-inflammatory food agents such as vegetables, fruits, nuts and fish, has an important effect on inflammatory response and consequently on IBD^6,7^.

Previous investigations documented that IBD patients are drastically exposed to high risk of osteopenia, osteoporosis and low-trauma fractures^8,9^. A case–control study conducted on 187 IBD patients and 420 healthy controls illustrated that IBD patients had less dietary calcium (Ca) intake than healthy controls. It suggested that dietary Ca intake of IBD patients is less than the recommended daily intake^10^. A prospective cohort study, that investigated a large European population (n = 401,326), could not find a significant relationship between dietary Ca intake and risk of UC or CD. However, milk consumers compared to non-consumers had a decreased risk of CD^11^. As far as we know, there is no study that has assessed this association in the Iranian population. Therefore, we aimed to investigate the relation between dietary Ca intake and UC prevalence in Iranian adults.

## Materials and methods

### Study participants

Our case–control study included UC outpatients and healthy controls who accepted to take part in the study. Diagnosed patients with UC by gastroenterologists that had been registered in the Iranian IBD registry were included as cases. Newly diagnosed patients with IBD had participated in an educational class to learn how they could control their stress and modify their lifestyle to decrease the disease symptoms. We used this class as an opportunity to invite IBD patients (n = 140) to our study and to aware them of our study design and its aims. Finally, 109 patients participated. There was no variety in general characteristics including age, physical activity, and residence area between those who agreed to participate, and those who did not. The data collection was applied from 2015 to 2019 in Isfahan, Iran. Data of Study on the Epidemiology of Psychological, Alimentary Health and Nutrition (SEPAHAN) were used to select healthy controls. More information about the SEPAHAN project is presented elsewhere^12^. This population-based study was conducted on a large population with more than 8000 apparently healthy individuals. First, we excluded patients with gastrointestinal disorders (including Crohn’s disease, ulcerative colitis, irritable bowel syndrome, functional dyspepsia, gastro-esophageal reflux disorder) from the SEPAHAN dataset. Then, two age (± 2 years) and sex-matched controls for each case were randomly chosen from healthy participants of the SEPAHAN dataset.

The study procedure was performed according to the declaration of Helsinki and the STROBE checklist. All participants provided informed written consent. The study protocol was approved by the local Ethics Committee of Isfahan University of Medical Sciences (Ethical number: IR.MUI.RESEARCH.REC.1400.341).

### Assessment of calcium intakes

Dietary intakes of participants were examined through the use of a validated self-administrated dish-based food frequency questionnaire (DS-FFQ). Details of this questionnaire are presented elsewhere^13^. In brief, using the Harvard FFQ as a model, a DS-FFQ was created. Several steps, including: construction of a list of commonly consumed Iranian foods, definition of portion sizes, determining the frequency response options for each food item, were taken to develop this DS-FFQ. Then, a pilot study was conducted to test the face validity of the FFQ. This face validity helped us to select appropriate number of response options for each food item in the list. This tool was examined five domains of raw and cooked foods and dishes (mixed dishes, potatoes and grain-based foods, dairy products, fruits and vegetables, miscellaneous foods and beverages). The participants were asked to determine the frequency of their intakes of 106 food items in 6–9 options, ranged from “never or less than once a month” to “12 or more times per day” in the last year. Then, the household measures were used to convert reported intakes to gram per day. Furthermore, the US Department of Agriculture (USDA) nutrient database was used for calculation of nutrients, including calcium intake per day. Additionally, the content of some Iranian foods based on the available Iranian food composition table was added to this database. Our validation study indicated that this questionnaire had appropriate levels of validity and reliability and could reasonably estimate long-term dietary intakes of Iranian adults^13^. As qualitative supports for the validity of this questionnaire, several relationships between dietary intakes and diseases have also established by the use of this DS-FFQ^14–16^.

### Assessment of ulcerative colitis

An expert gastroenterologist diagnosed patients with UC based on international criteria^17^ including, colonoscopy, physical and histological examinations. Additionally, medical records were reviewed to confirm the diagnosis.

### Assessment of covariates

A self-administered questionnaire was used for assessment of demographic characteristics and medical history of participants including age, sex (male/female), education (high school graduated or below/academic education), marital status (single/married), family size (≤ 4/> 4 members), smoking status (yes/no), houseownership (yes/no), and medical history including the existence of gallstone, hypertension, hyperlipidemia, Crohn’s disease, and type 2 diabetes. Data of anthropometric variables were also collected by using a validated self-administered questionnaire^18^. Body mass index (BMI) was calculated as weight (kg) divided by height squared (m^2^). Our previous study had shown that the applied questionnaire provided valid information in comparison to actual measured values^18^. Physical activity of the study population was also examined through the use of the General Practice Physical Activity Questionnaire (GPPAQ). Based on this questionnaire, the physical activity of participants was categorized as no activity, < 3 h activity per week, 3–5 h per week, 5–7 h per week, and ≥ 7 h activity per week.

### Statistical analysis

We computed the required sample size for this study based on a previous published investigation which showed that approximately 60% of Iranian adults have inadequate calcium intake^19^. Furthermore, based on previous publications, the risk of IBD would be double among individuals with inappropriate dietary intakes^20^. Therefore, considering the study power of 80%, type I error of 5%, and 2 controls per case, at least 101 cases and 202 controls were required to include in the current study. We categorized both cases and controls together based on tertiles of energy adjusted-dietary Ca intake including, T_1_: < 695 mg/day, T2: 695–983 mg/day, and T3: > 983 mg/day. The distribution of participants in terms of categorical and continuous variables across tertiles of energy adjusted-dietary Ca intake was respectively determined through the use of the chi-square test and one-way analysis of variance (ANOVA). Analysis of covariance (ANCOVA) was also applied to achieve energy, age, and sex-adjusted dietary intakes of subjects across tertiles of energy adjusted-dietary Ca intake. The odds (ORs) of UC across the tertiles of energy adjusted-dietary Ca intake were examined through the use of binary logistic regression, in crude and multivariable-adjusted models. In addition, we examined the odds of UC based on the recommended dietary allowances (RDAs) for Ca (Males: ≥ 1000 mg; Women: 19–50 years: ≥ 1000 mg; ≥ 51 years: ≥ 1200 mg) based on an updated recommendation^21^. We selected potential confounders related to IBD and UC based on earlier investigations^22,23^. In the first model, we controlled for age (continuous), sex (male/female), and energy intake (continuous). In the second model, we additionally made adjustments for marital status (single/married), education (high school graduated or below/academic education), family size (≤ 4/> 4 members), smoking (yes/no), hypertension (yes/no), type 2 diabetes (yes/no), physical activity (no activity/< 3 h per week/3–5 h per week/5–7 h per week/> 7 h per week) and house ownership (yes/no). In the last model, body mass index (continuous) was additionally adjusted. Those in the first tertile of energy adjusted-dietary Ca intake were considered as the reference group in these analyses. In the analysis based on RDA, subjects with sufficient Ca intake were considered as the reference category. Energy adjusted tertiles of dietary Ca intake were treated as an ordinal variable in logistic regression models for determining P for trends. All analyses were performed through the use of SPSS software version 20. P-values were considered statistically significant at < 0.05.

### Ethical approval and consent to participate

The study procedure was performed according to declaration of Helsinki and STROBE checklist. All participants provided informed written consent. The study protocol was approved by the local Ethics Committee of Isfahan University of Medical Sciences (Ethical number: IR.MUI.RESEARCH.REC.1400.341).

## Results

### Characteristics of UC cases and healthy controls

**Figure 1:**
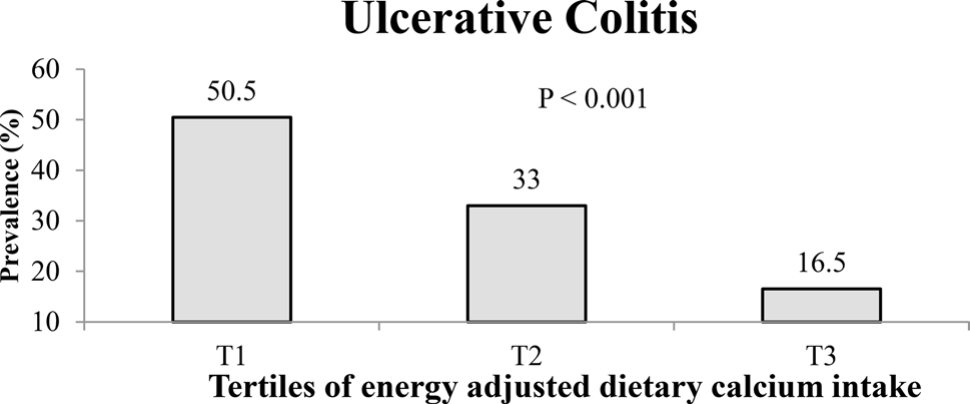
The prevalence of ulcerative colitis across tertiles of energy adjusted-dietary Ca intake.

Overall, we included 327 participants (109 cases and 218 controls) in the current analysis and 52.1% of them were females. The mean age of the total population was 40.17 ± 10.66 (years) and there were no significant age differences between UC cases (39.5 ± 10.0 years) and controls (41.5 ± 11.8 years). In addition, there was no significant difference in BMI or other variables (including sex, smoking status, marital status, and history of diabetes) between cases and controls. Nevertheless, the UC patients were less likely to be university graduated (38 vs. 54%; P = 0.007) and physically active (17 vs. 34%; P = 0.02). Mean intake of dietary Ca in whole population was 921.7 mg/day (± 447.9). Dietary Ca intake was lower in cases than controls (736.6 ± 315.4 vs. 1014.2 ± 475.5; P < 0.001). Moreover, inadequate dietary Ca intake was more prevalent among UC cases compared with healthy controls (78.0% vs. 66.5%; P = 0.02). In addition, when we distributed participants across tertiles of energy adjusted-dietary Ca intake, there were no significant differences in the demographic variables, except for the prevalence of hypertension which was significantly lower in the second tertile than other categories (**Table ****1**). The prevalence of UC in tertiles of energy adjusted-dietary Ca intake had a significant decreasing trend (P < 0.001); such that, 50.5, 33 and 16.5% of individuals in tertiles 1, 2 and 3 of energy adjusted-dietary Ca intake have respectively suffered from UC (**Fig. ****1**).

**Table 1:** Characteristics of study participants and their distribution across tertiles of energy adjusted-dietary calcium intake.

	Total participants	Tertiles of energy adjusted-dietary Ca intake			
	(n = 327)	T1 (n = 109) < 695 mg/day	T2 (n = 109) 695–983 mg/day	T3 (n = 109) > 983 mg/day	P*
Age (years) Mean ± SD	40.17 ± 10.66	40.40 ± 10.79	39.10 ± 10.74	41.01 ± 10.47	0.41
BMI (kg/m^2^) Mean ± SD	25.29 ± 3.73	25.31 ± 3.90	25.38 ± 3.69	25.18 ± 3.65	0.92
Sex (females), n (%)	170 (52.1)	50 (45.9)	59 (54.6)	61(56.0)	0.26
Marital status, (married) n (%)	256 (78.3)	83 (76.1)	84 (77.1)	89 (81.7)	0.57
Smoking status, (current smoker) n (%)	21 (7.1)	11 (10.8)	5 (5.1)	5 (5.2)	0.35
History of type 2 diabetes (yes) n (%)	9 (2.8)	3 (2.8)	2 (1.9)	4 (3.7)	0.71
History of hypertension (yes) n (%)	22 (6.7)	10 (9.2)	2 (1.9)	10 (9.2)	**0.04**
Education, (university graduated) n (%)	156 (48.4)	51 (46.8)	50 (47.6)	55 (50.9)	0.81
Family size, (> 4 members) n (%)	33 (10.1)	15 (13.8)	6 (5.6)	12 (11.0)	0.12
House ownership, (yes) n (%)	212 (65.6)	73 (68.2)	72 (67.3)	67 (61.5)	0.77
Physical activity, n (%)					0.65
No activity	17 (7.3)	7 (9.6)	7 (9.1)	3 (3.7)	
< 3 h per week	72 (31.0)	26 (35.6)	20 (26.0)	26 (31.7)	
3–5 h per week	54 (23.3)	13 (17.8)	18 (23.4)	23 (28.0)	
5–7 h per week	26 (11.2)	7 (9.6)	9 (11.7)	10 (12.2)	
≥ 7 h per week	63 (27.2)	20 (27.4)	23 (29.9)	20 (24.4)	

Significant values are in [bold].

*Obtained from ANOVA or chi-squared test, where appropriate.

### Dietary intakes of participants across tertiles of energy adjusted-dietary Ca intake

Details of dietary intakes of energy and selected nutrients across tertiles of energy adjusted-dietary Ca intake are presented in **Table ****2**. Individuals in the highest tertile of energy adjusted-dietary Ca intake in comparison to the lowest tertile reported significantly higher dietary intake of carbohydrates, dietary fiber, vitamin B_6_, niacin, riboflavin, thiamin, vitamin C, iron, and magnesium. In contrast, participants in the top tertile of energy adjusted-dietary Ca intake had a significantly lower intake of energy, total fats, monounsaturated fatty acids (MUFA), polyunsaturated fatty acids (PUFA) and vitamin B_12_, compared with those in the bottom tertile. However, there was no significant difference in dietary intakes of proteins, SFA, folate, vitamin A, vitamin E and zinc, across tertiles of energy adjusted-dietary Ca intake.

**Table 2:** Dietary intakes of selected nutrients for study participants across tertiles of energy adjusted-dietary Ca intake.

	Tertiles of energy adjusted-dietary Ca intake						
	T1 (n = 109) < 695 mg/day		T2 (n = 109) 695–983 mg/day		T3 (n = 109) > 983 mg/day		P*
	Mean	SE	Mean	SE	Mean	SE	
Energy (kcal/day)	2631.52	95.99	2210.33	94.17	2590.34	94.05	**0.003**
**Nutrients**							
Carbohydrates (%)	45.00	0.86	47.26	0.85	51.906	0.84	**< 0.001**
Proteins (%)	14.67	0.26	14.74	0.26	15.25	0.26	0.23
Total fats (%)	41.02	0.69	39.03	0.68	34.13	0.67	**< 0.001**
Dietary fiber (g/day)	18.72	0.60	21.27	0.60	22.92	0.59	**< 0.001**
MUFA (g/day)	38.04	1.02	37.67	1.01	33.43	1.00	**0.002**
PUFA (g/day)	40.70	1.07	36.08	1.06	27.09	1.05	**< 0.001**
SFA (g/day)	24.24	0.57	24.98	0.56	24.04	0.56	0.47
Vitamin B12 (mcg/day)	5.29	0.40	4.76	0.40	3.78	0.39	**0.02**
Vitamin B6 (mg/day)	1.78	0.04	1.99	0.04	1.94	0.04	**0.005**
Folate food (mcg/day)	354.581	10.43	367.18	10.32	354.85	10.20	0.62
Niacin (mg/day)	24.33	0.48	23.98	0.47	25.62	0.47	**0.03**
Riboflavin (mg/day)	1.46	0.03	1.79	0.03	2.29	0.03	**< 0.001**
Thiamine (mg/day)	1.67	0.05	1.75	0.05	2.17	0.05	**< 0.001**
Vitamin A (RE/day)	684.58	39.14	743.82	38.72	630.71	38.28	0.12
Vitamin C (mg/day)	95.75	5.93	116.45	5.87	118.08	5.80	**0.01**
Vitamin E (mg/day)	6.51	0.74	8.53	0.91	5.54	1.27	0.11
Fe (mg/day)	16.98	0.32	17.06	0.31	18.83	0.31	**< 0.001**
Mg (mg/day)	289.86	6.08	331.38	6.01	352.45	5.95	**< 0.001**
Zn (mg/day)	11.38	0.16	11.81	0.16	11.83	0.16	0.09

Significant values are in [bold].

*All values were adjusted for age, sex and energy; except dietary energy intake, which was only adjusted for age and sex by using ANCOVA.

### Association of dietary Ca intake with UC

Multivariable-adjusted odds ratios and 95% CIs for UC across categories of dietary Ca intake are shown in **Table ****3**. Participants in the top tertile of energy Ca intake had lower odds of UC, compared to those in the bottom tertile (OR = 0.19, 95% CI 0.10–0.36). After adjustments for potential confounders, this relation was strengthened; such that, individuals in the third tertile of dietary Ca intake had 92% lower odds of UC, in comparison to those in the first tertile. In addition, odds of UC in tertiles of dietary Ca intakes had a significant decreasing trend (P-trend < 0.001). Our analysis based on RDA intake showed that dietary Ca intake deficiency was related to increased odds of UC (OR = 1.78, 95% CI 1.05–3.04). This association became stronger after adjustment for all confounders; in such a way that, individuals with inadequate dietary Ca intake had 9.5 times higher odds of UC, in comparison to participants with sufficient Ca intake (OR: 9.5, 95% CI 2.98–30.91). Our stratified by gender showed that in females, the highest dietary Ca intake was related to 89% lower odds of UC, compared to the lowest intake (OR: 0.11, 95% CI 0.02–0.56) (**Table ****4**). Insufficient dietary Ca intake in females was also related to 13.99 times higher odds of UC, compared to those with sufficient Ca intake (OR: 13.99, 95% CI 2.47–79.04). These associations were stronger in the male population. So that, males in the top tertile of dietary Ca intake, in comparison to those in the bottom category, had 93% decreased odds of UC (OR: 0.07, 95% CI 0.01–0.39) (**Table ****4**). Furthermore, males with inadequate dietary Ca intake had 16 times higher odds of UC, in comparison to those with sufficient intake (OR: 16.00, 95% CI 2.38–107.32). Moreover, the odds of UC in tertiles of dietary Ca intakes had a significant decreasing trend in both males and females (P-trend = 0.007 and 0.002, respectively).

**Table 3:** Multivariable- adjusted odds ratio and 95% confidence intervals for ulcerative colitis (UC) across tertiles of energy adjusted-dietary Ca intake.

	Tertiles of energy adjusted-dietary Ca intake			P-trend	Based on RDA^a^	
	T1 (n = 109) < 695 mg/day	T2 (n = 109) 695–983 mg/day	T3 (n = 109) > 983 mg/day		Sufficient intake (n = 97)	Under RDA (n = 230)
Cases (n)	55	36	18		24	85
Crude	1 (Ref)	0.48 (0.28, 0.83)	0.19 (0.10, 0.36)	< 0.001	1 (Ref)	1.78 (1.05, 3.04)
Model 1^b^	1 (Ref)	0.56 (0.31, 1.03)	0.18 (0.09, 0.35)	< 0.001	1 (Ref)	4.71 (2.33, 9.5)
Model 2^c^	1 (Ref)	0.42 (0.17, 1.05)	0.07 (0.02, 0.23)	< 0.001	1 (Ref)	8.48 (2.82, 25.46)
Model 3^d^	1 (Ref)	0.44 (0.17, 1.14)	0.08 (0.02, 0.27)	< 0.001	1 (Ref)	9.5 (2.98, 30.91)

All values are odds ratios and 95% confidence intervals.

^a^RDA: Recommended Dietary Allowances for dietary Ca intake (Man: ≥ 1000 mg; Women: 19–50 years: ≥ 1000 mg; ≥ 51 years: ≥ 1200 mg).

^b^Model 1: Adjusted for age, sex, and energy intake.

^c^Model 2: Additionally adjusted for marital status, education, family size, smoking, hypertension, diabetes, physical activity and house ownership.

^d^Model 3: Further adjusted for body mass index (BMI).

**Table 4:** Multivariable-adjusted odds ratio and 95% confidence intervals for ulcerative colitis (UC) across tertiles of energy adjusted-dietary Ca intake, stratified by gender.

	Tertiles of energy adjusted-dietary Ca intake			P-trend	Based on RDA^a^	
	T1	T2	T3		Sufficient intake	Under RDA
**Female**						
Participants/cases (n)	50/24	59/21	61/11		46/11	124/45
Crude	1 (Ref)	0.59 (0.27, 1.29)	0.23 (0.10, 0.56)	0.001	1 (Ref)	1.81 (0.83, 3.91)
Model 1^b^	1 (Ref)	0.63 (0.28, 1.41)	0.20 (0.08, 0.50)	0.001	1 (Ref)	3.46 (1.31, 9.12)
Model 2^c^	1 (Ref)	0.41 (0.11, 1.50)	0.09 (0.02, 0.42)	0.002	1 (Ref)	16.39 (2.81, 95.40)
Model 3^d^	1 (Ref)	0.46 (0.11, 1.82)	0.11 (0.02, 0.56)	0.007	1 (Ref)	13.99 (2.47, 79.04)
**Male**						
Participants/cases (n)	59/31	49/14	48/7		51/13	105/39
Crude	1 (Ref)	0.36 (0.16, 0.80)	0.15 (0.06, 0.39)	< 0.001	1 (Ref)	1.72 (0.82, 3.63)
Model 1^b^	1 (Ref)	0.49 (0.20, 1.21)	0.16 (0.06, 0.44)	< 0.001	1 (Ref)	7.07 (2.41, 20.75)
Model 2^c^	1 (Ref)	0.52 (0.13, 2.05)	0.06 (0.01, 0.35)	0.002	1 (Ref)	9.23 (1.80, 47.24)
Model 3^d^	1 (Ref)	0.51 (0.12, 2.06)	0.07 (0.01, 0.39)	0.002	1 (Ref)	16.0 (2.38, 107.32)

All values are odds ratios and 95% confidence intervals.

^a^RDA: Recommended Dietary Allowances for dietary Ca intake (Man: ≥ 1000 mg; Women: 19–50 years: ≥ 1000 mg; ≥ 51 years: ≥ 1200 mg).

^b^Model 1: Adjusted for age, and energy intake.

^c^Model 2: Additionally adjusted for marital status, education, family size, smoking, hypertension, diabetes, physical activity and house ownership.

^d^Model 3: Further adjusted for body mass index (BMI).

## Discussion

We found that higher intake of dietary Ca, with a decreasing trend, was associated with lower odds of UC in Iranian adults. In addition, inadequate dietary Ca intake was drastically related to higher odds of UC in comparison to sufficient intake. These associations were strengthened, after considering the effect of potential confounders.

The prevalence of UC has been drastically increased over recent decades, in developing countries. Due to the negative effects of UC on social function, mental health and public health, this disease could impose remarkable costs on healthcare systems^22^. Based on previous evidence, IBD patients had low bone mineral density (BMD), because of steroids therapy in patients with severe disease and/or frequent relapses^24,25^. Some investigations illustrated that long-term calcium and vitamin D supplementation could not considerably improve BMD in healthy subjects^26^ and patients with IBD on corticosteroid therapy^27^. Whereas, sufficient dietary intake of calcium and vitamin D would have a favorable impact on calcium balance and alter bone metabolism, both in children and in adults^28–31^.

In the current study, a significant inverse association was observed between dietary Ca intake and UC in adults. Additionally, deficient Ca intake was positively associated with UC in both men and women. In line with our findings, a case–control study that considered both CD and UC as IBD patients in Italian subjects demonstrated that IBD patients had significantly less dietary calcium intake than healthy controls. Calcium intake in this investigation was lower in the oldest IBD patients and in female IBD patients. In addition, 37.8% of IBD patients had adequate (> 100% RDA) daily calcium intake, while 35.0% had extremely low intake (< 70% RDA)^10^. Moreover, a prospective study on the European population indicated that milk consumers compared with non-consumers had a decreased risk for developing CD, although the relation of dietary Ca intake or dairy product with risk of CD was not significant^11^. In the current analysis, just UC patients were included as IBD cases. Our investigation revealed that inadequate dietary Ca intake was drastically related to higher odds of UC, compared to patients with sufficient Ca intake. In addition, the prevalence of UC was significantly lower in the top tertile of dietary Ca intake, compared with the bottom tertile.

The most important reason for inadequate dietary Ca intake in IBD patients could be the belief that dairy products, especially milk, could exacerbate the disease symptoms. Some physicians might recommend IBD patients who suffer from diarrhea, to avoid or restrict their dairy products intake^32–34^. However, it should be considered that the lactose content of dairy products other than milk (such as yogurt and cheese) is minimal and they are good sources of dietary Ca intake. Furthermore, some previous investigations indicated that intestinal inflammation could be reduced through two main pathways. First, consumption of dairy products, especially fermented milk products, could increase the abundance of bacteria-producing butyrate, an important colonic energy source and consequently interfere with the colitogenic environment^35–38^. Second, consumption of fortified milk with vitamin D would modulate the inflammation in IBD patients, because vitamin D status could drastically affect IBD^39,40^.

The present study has some strengths and weaknesses. As far as we know, this is the first population-based case–control study on Iranian adults that investigated the UC prevalence in relation to dietary Ca intake. Additionally, a validated FFQ was used for the assessment of dietary intakes of participants. In statistical analysis, several potential confounders (including main confounders (such as age, sex, physical activity, and energy intake), socio-demographic variables, medical history of participants and BMI) were also taken into account, to obtain an independent relationship from potential confounders and obesity. However, some limitations must be kept in mind while interpreting the findings. We could not determine the causality, due to the case–control design of the study; so, prospective investigations should confirm the causality of this relation. Furthermore, recall bias and selection bias, as two inevitable sources of bias in case–control studies, might affect the results. In addition, misclassification or measurement errors for dietary Ca intake (as exposure) might happen, although a validated FFQ was used for this assessment. Since using dietary supplements is not common in middle-aged Iranian adults, we did not gather information of taking Ca supplement from the study subjects. This limitation might also lead to misclassification of participants in terms of the exposure of interest. Furthermore, cases were not newly diagnosed patients; therefore, their dietary intakes might be changed as a result of their disease conditions. Finally, there was no precise information about the duration of disease in UC cases; so, we could not control the effect of this covariate in the analysis.

## Conclusions

The current population-based case–control study revealed that higher dietary calcium intake was associated with lower UC prevalence in Iranian adults. Inadequate dietary calcium intake was also linked to elevated likelihood of UC. Further investigations, especially with prospective design, are needed to affirm these findings.

## Data Availability

Data of Study on the Epidemiology of Psychological, Alimentary Health and Nutrition (SEPAHAN) were used to select healthy controls. The datasets used and analyzed during the current study are available from the corresponding author (Dr. Parvane Saneei at email: saneeip@yahoo.com) on reasonable request.

## Abbreviations

IBD: Inflammatory bowel disease
CD: Crohn's disease
UC: Ulcerative colitis
BMI: Body mass index
OR: Odds ratio
95% CI: 95% Confidence interval
DS-FFQ: Dish-based food frequency questionnaire
USDA: US Department of Agriculture
GPPAQ: General Practice Physical Activity Questionnaire
RDAs: Recommended dietary allowances
BMD: Bone mineral density
SEPAHAN: Study on the Epidemiology of Psychological-Alimentary Health and Nutrition
IUMS: Isfahan University of Medical Sciences
ANOVA: Analysis of variance
ANCOVA: Analysis of covariance
SPSS: Statistical package for the social sciences
SD: Standard deviation
SE: Standard error

## References

[1] Rogler G, Biedermann L, Scharl M (2018). New insights into the pathophysiology of inflammatory bowel disease: Microbiota, epigenetics and common signalling pathways. Swiss Med. Wkly..

[2] Zhou, M. *et al.* New frontiers in genetics, gut microbiota, and immunity: a rosetta stone for the pathogenesis of inflammatory bowel disease. *BioMed Res. Int.* **2017**, 8201672 (2017). 10.1155/2017/8201672 PMC5558637 28831399

[3] Malekzadeh, M. M. *et al.* Emerging epidemic of inflammatory bowel disease in a middle income country: a nation-wide study from Iran. *Arch. Iran. Med.* **19**, 2–15 (2016). 26702742

[4] Asakura H, Suzuki K, Kitahora T, Morizane T (2008). Is there a link between food and intestinal microbes and the occurrence of Crohn's disease and ulcerative colitis?. J. Gastroenterol. Hepatol..

[5] Ruemmele FM (2016). Role of diet in inflammatory bowel disease. Ann. Nutr. Metab..

[6] Khalili H (2020). Adherence to a Mediterranean diet is associated with a lower risk of later-onset Crohn’s disease: Results from two large prospective cohort studies. Gut.

[7] Vrdoljak J (2020). Mediterranean diet adherence and dietary attitudes in patients with inflammatory bowel disease. Nutrients.

[8] Targownik LE, Bernstein CN, Leslie WD (2013). Inflammatory bowel disease and the risk of osteoporosis and fracture. Maturitas.

[9] Ali T, Lam D, Bronze MS, Humphrey MB (2009). Osteoporosis in inflammatory bowel disease. Am. J. Med..

[10] Vernia P (2014). Dietary calcium intake in patients with inflammatory bowel disease. J. Crohns Colitis.

[11] Opstelten JL (2016). Dairy products, dietary calcium, and risk of inflammatory bowel disease: Results from a European prospective cohort investigation. Inflamm. Bowel Dis..

[12] Adibi P (2012). The study on the epidemiology of psychological, alimentary health and nutrition (SEPAHAN): Overview of methodology. J. Res. Med. Sci..

[13] Keshteli AH (2014). A dish-based semi-quantitative food frequency questionnaire for assessment of dietary intakes in epidemiologic studies in Iran: Design and development. Int. J. Prev. Med..

[14] Tabibian, S. R. *et al.* The relationship between fruit and vegetable intake with functional dyspepsia in adults. *Neurogastroenterol Motil*. **33**, e14129 (2021). 10.1111/nmo.14129 33797127

[15] Saneei, P. *et al.* Adherence to alternative healthy eating index in relation to depression and anxiety in Iranian adults. *Br J Nutr*. **116**, 335–342 (2016). 10.1017/S0007114516001926 27188471

[16] Saneei, P. *et al.* Adherence to the DASH diet and prevalence of the metabolic syndrome among Iranian women. *Eur J Nutr.* **54**, 421–428 (2015). 10.1007/s00394-014-0723-y 24906470

[17] Lennard-Jones J (1989). Classification of inflammatory bowel disease. Scand. J. Gastroenterol..

[18] Aminianfar A (2021). Validity of self-reported height, weight, body mass index, and waist circumference in Iranian adults. Int. J. Prev. Med..

[19] Hosseinzadeh P (2019). The association of dietary intake of calcium and vitamin d to colorectal cancer risk among Iranian population. Asian Pacif. J. Cancer Prevent. APJCP.

[20] Shivappa N, Hébert JR, Rashvand S, Rashidkhani B, Hekmatdoost A (2016). Inflammatory potential of diet and risk of ulcerative colitis in a case–control study from Iran. Nutr. Cancer.

[21] Raymond JL, Morrow K (2020). Krause and Mahan’s Food and the Nutrition Care Process E-Book.

[22] Windsor JW, Kaplan GG (2019). Evolving epidemiology of IBD. Curr. Gastroenterol. Rep..

[23] Cui G, Yuan A (2018). A systematic review of epidemiology and risk factors associated with Chinese inflammatory bowel disease. Front. Med..

[24] Schulte C, Dignass AU, Mann K, Goebell H (1998). Reduced bone mineral density and unbalanced bone metabolism in patients with inflammatory bowel disease. Inflamm. Bowel Dis..

[25] Tilg H, Moschen A, Kaser A, Pines A, Dotan I (2008). Gut, inflammation and osteoporosis: Basic and clinical concepts. Gut.

[26] Uusi-Rasi K, Kärkkäinen MU, Lamberg-Allardt CJ (2013). Calcium intake in health maintenance—A systematic review. Food Nutr. Res..

[27] Siffledeen JS (2005). Randomized trial of etidronate plus calcium and vitamin D for treatment of low bone mineral density in Crohn’s disease. Clin. Gastroenterol. Hepatol..

[28] Gennari C (2001). Calcium and vitamin D nutrition and bone disease of the elderly. Public Health Nutr..

[29] Huncharek M, Muscat J, Kupelnick B (2008). Impact of dairy products and dietary calcium on bone-mineral content in children: Results of a meta-analysis. Bone.

[30] Welten DC, Kemper HC, Post GB, Van Staveren WA (1995). A meta-analysis of the effect of calcium intake on bone mass in young and middle aged females and males. J. Nutr..

[31] Esterle L, Nguyen M, Walrant-Debray O, Sabatier JP, Garabedian M (2010). Adverse interaction of low-calcium diet and low 25 (OH) D levels on lumbar spine mineralization in late-pubertal girls. J. Bone Miner. Res..

[32] Wright R, Truelove S (1965). A controlled therapeutic trial of various diets in ulcerative colitis. BMJ.

[33] Bernstein, C. N., Ament, M., Artinian, L., Ridgeway, J. & Shanahan, F. Milk tolerance in adults with ulcerative colitis. *Am. J. Gastroenterol. (Springer Nat.)*. **89**, 872–877 (1994). 8198097

[34] Ballegaard M (1997). Self-reported food intolerance in chronic inflammatory bowel disease. Scand. J. Gastroenterol..

[35] Da Silva MS, Rudkowska I (2015). Dairy nutrients and their effect on inflammatory profile in molecular studies. Mol. Nutr. Food Res..

[36] Labonte M-E, Couture P, Richard C, Desroches S, Lamarche B (2013). Impact of dairy products on biomarkers of inflammation: A systematic review of randomized controlled nutritional intervention studies in overweight and obese adults. Am. J. Clin. Nutr..

[37] Veiga P (2010). *Bifidobacterium animalis* subsp. lactis fermented milk product reduces inflammation by altering a niche for colitogenic microbes. Proc. Natl. Acad. Sci..

[38] SantosRocha C (2012). Anti-inflammatory properties of dairy lactobacilli. Inflamm. Bowel Dis..

[39] Ananthakrishnan AN (2012). Higher predicted vitamin D status is associated with reduced risk of Crohn's disease. Gastroenterology.

[40] Palmer MT, Weaver CT (2013). Linking vitamin d deficiency to inflammatory bowel disease. Inflamm. Bowel Dis..

